# ASTHMAXcelED: A Patient-Facing Asthma Mobile Application with High User Acceptance and Improved Clinical Outcomes

**DOI:** 10.1055/a-2812-0739

**Published:** 2026-03-06

**Authors:** Carlo Lutz, Arvind Haran, Sonia Sakleshpur, Nicholas Santavicca, Eddie Irizzary, Andrew Williams, Joshua Kaiser, Shilpa Kolli, Sunit Jariwala, Benjamin W. Friedman

**Affiliations:** 1 Department of Emergency Medicine, Montefiore Medical Center/Albert Einstein College of Medicine, Bronx, New York, United States; 2 Department of Medicine, Division of Allergy and Immunology, Montefiore Medical Center/Albert Einstein College of Medicine, Bronx, New York, United States

**Keywords:** asthma, mobile application, emergency medicine, health education

## Abstract

**Background:**

Well-designed mobile health (mHealth) applications can improve patient outcomes; however few clinically tested apps are developed with user-centric design. ASTHMAXcelED is a patient-facing mobile application designed for emergency department (ED) asthma patients.

**Objectives:**

We studied user acceptance of the application and compared asthma control and quality of life measures before and after use of ASTHMAXcelED.

**Methods:**

This prospective cohort study was conducted in two large urban EDs. Inclusion criteria were patients ≥18 years, English literacy, smartphone access, and discharge from the ED diagnosed with asthma exacerbation. ASTHMAXcelED was downloaded onto participants' phones upon discharge. Asthma symptom control was measured using the Asthma Control Test (ACT). User acceptance of and intention to use the application after the study period was measured using a Unified Theory of Acceptance and Use of Technology.

**Results:**

A total of 149 participants were enrolled in the study, of which 124 completed follow-up visits. The proportion of patients with good asthma control (ACT > 19) improved from 27% at baseline to 54% at 4-week follow-up, *p* < 0.01. The rate of ED revisit was 7% and hospitalization for asthma was 0%. A total of 39% of patients indicated they intended to continue using ASTHMAXcelED in the next 6 months, whereas 5% did not.

**Conclusion:**

While using ASTHMAXcelED for 4 weeks, participant's asthma symptom control and quality of life scores improved. Their ED revisit rate and asthma hospitalization during the study period were low. ASTHMAXcelED may be an easily adopted mhealth education platform that can improve patient outcomes.

## Background and Significance

Asthma accounts for 2 million emergency department (ED) visits and 500,000 hospitalizations in the United States annually, with over $2 billion spent on ED asthma visits. 1 The Bronx has the highest asthma burden in New York State and one of the highest in the country, with hospitalization and ED visit rates triple the state average. 2 The borough is the most socioeconomically disadvantaged in New York City and its population consists of a majority of racial and ethnic minorities; 3 demographic groups with worse asthma morbidity than other groups. 4 Low health literacy, 5 6 7 poor health care access, 8 exposure to asthma triggers, 9 and poor medication adherence 10 11 are known contributors to increased asthma burden and are more common in these populations.

Asthma self-management education improves health outcomes through medication adherence and trigger identification. 12 ED-based education is necessary because many patients who visit the ED for asthma see it as their preferred setting for asthma, regardless of access to alternative sources of care or even the severity of their asthma. 13 However, despite American Thoracic Society recommendations, patients discharged from the ED after treatment for asthma exacerbations rarely receive comprehensive guideline-based asthma education or a specific discharge plan. 14 Asthma education in the ED has high feasibility barriers, due to an already high burden of health care professionals' responsibilities, competing priorities, and rapid staff turnover. 15 16

Smartphone-based platforms designed to improve asthma self-management can improve patient-centered outcomes in asthma. 17 High rates of smartphone ownership even among socioeconomically disadvantaged populations 18 and the acceptance of mobile health (mHealth) technologies by the public, as illustrated by the exponential growth in health/fitness app usage, 19 makes mHealth apps a powerful, scalable health care solution. However, few studies have investigated their utility in the ED population. Many mobile applications, defined here as “self-contained software designed for a mobile device and performing specific tasks for mobile users,” 20 fail to provide high-quality, guideline-directed, comprehensive information for patients. Published literature suggests that 100 of 103 apps lacked adequate information, and 50% of the recommendations in these applications are not in line with current guidelines. 21 Many applications are also poorly received by users, who described most asthma-related applications as having poor functionality and usability, 22 23 which limits sustained user engagement and the ability of those apps to promote improved health outcomes. 24

The original ASTHMAXcel mobile application provides asthma education and promotes adherence to national asthma guidelines. Over the past decade, this platform has been iteratively improved with patient feedback in line with a user centered design approach. 24 ASTHMAXcel has been shown to improve asthma knowledge, asthma control, and asthma-related quality of life (QoL) among asthma clinic patients. 17 Building on this success, ASTHMAXcelED was designed to specifically address common issues ED asthma patients face, particularly medication adherence 10 11 and trigger avoidance. 9 This version is intended for self-guided learning with fewer, simpler modules, and tailored biweekly push notifications emphasizing best practices. While all users receive instruction on proper inhaler technique and recognizing environmental triggers, ASTHMAXcelED teaches newly diagnosed asthma patients about basic asthma physiology and proper medication use and assists patients with more established asthma diagnoses in developing an asthma action plan.

## Objectives

This study investigates if patients discharged from the ED with asthma exacerbations who use ASTHMAXcelED application have improved asthma symptom control and asthma-related QoL and evaluated their experience with the application. We hypothesized that participants' asthma symptom control and QoL would have sustained improvement 4 weeks after discharge. We also hypothesized that users would have high intention to use the application in the future and would be correlated with user expectations regarding application performance, ease of use, and social influences.

## Methods

### Participants

This prospective cohort study took place at two Bronx urban EDs from May 2021 to March 2022. ED clinicians referred patients aged 18 and over with asthma exacerbations to in-house research associates (RAs) for enrollment. RAs screened participants just prior to their discharge from the ED for the following inclusion criteria: a self-reported history of physician-diagnosed asthma; ED discharge diagnosis of asthma exacerbation; English literacy; and smartphone ownership. RAs, under the guidance of the attending ED physician, excluded patients with severe cognitive or psychiatric conditions that precluded them from understanding or completing study participation. RAs downloaded the ASTHMAXcelED application onto participants' smartphones and demonstrated how to use the application at the time of enrollment. Participants enabled push notification functionality on their smartphones when initially downloading the application. Demographic and health information was also obtained at the time of enrollment. Data were then entered into our project's secure REDCap database and participants were scheduled for a 2-week follow-up call to administer a survey measuring asthma QoL as well as a 4-week follow-up call to administer surveys regarding asthma control and app-related user feedback. Participants did not receive compensation for their participation. This study was approved by the Albert Einstein College of Medicine Institutional Review Board and all subjects provided informed consent. This prospective trial was registered with the *ClinicalTrials.gov* and the trial registration number is NCT05013073.

### ASTHMAXcelED Mobile Application

The ASTHMAXcelED mobile application was developed for iOS and Android smartphone and tablet devices as an interactive mobile application. The material is divided into nine chapters, each featuring animated educational videos of 1 to 2 minutes in length, followed by a self-assessment quiz with questions on the topics presented. Each chapter is based on the National Asthma Education and Prevention Program guidelines, 25 the 2019 British Thoracic Society and Scottish Intercollegiate Guidelines Network guidelines, 26 and the 2018 Global Initiative for Asthma guidelines. 27 To ensure consistency with these guidelines, educational content was developed and vetted by asthma physicians, educators, and a behavioral scientist.

**Fig. 1:**
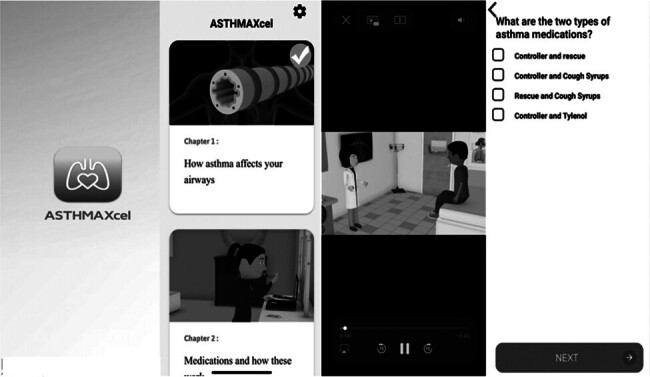
Screenshots of ASTHMAXcelED's Interface.

The content focused on medication adherence and trigger avoidance, which are established factors associated with asthma exacerbation in these patients. 4 10 11 17 24 The application also guides users in development of an asthma action plan, an evidence-based practice. Screenshots of the ASTHMAXcelED interface can be seen in Fig. 1 . 14 Users also receive biweekly push notifications regarding asthma-related best practices. These brief, 1-sentence notifications include statements such as “Use the controller medication every day and rescue medication when you need immediate relief,” and “If you have central air ventilation, put filters on all the vents!” The timing of these notifications is based on feedback from previous iterations of the ASTHMAXcel application.

### Measures

#### Asthma Control Test and Health Care Utilization

The primary outcome, asthma symptom control at 4 weeks, was measured using the Asthma Control Test (ACT). ACT is a validated tool that assesses general asthma symptoms, rescue medication use, functional status, and general asthma self-assessment over a 4-week recall period. 28 The survey includes five questions scored on a 5-point Likert scale, summed to calculate the ACT score. Potential ACT scores range from 5 (poor control to asthma) to 25 (perfect control of asthma), with a score >19 indicating good asthma control. The minimum clinically relevant difference is three points between two groups or over time. 28

Additionally, RAs asked participants if they had presented to any ED for asthma or been admitted to the hospital for asthma during the study period.

#### Quality of Life

To assess ASTHMAXcelED's effect on asthma-related QoL 29 we asked users the following questions:

1. How often do you feel short of breath?
2. How often are you bothered by dust in the environment?
3. How often do you wheeze?
4. How often do you have to avoid weather or air pollution?
5. How often are you limited by strenuous activities?

Each of these questions were scored on a 7-point Likert scale with 1 corresponding with “all the time” and 7 with “none of the time.” The survey was administered at discharge as well as 2 and 4 weeks after discharge from the ED, to see if QoL measures would continue to improve between 2 and 4 weeks.

#### User Acceptance and Intention to Use

Venkatesh et al. incorporated several human acceptance and behavior models into the Unified Theory of Acceptance and Use of Technology (UTAUT). 30 The model explained 70% of the variance in Behavioral Intention to Use (BI) and about 50% in actual use 31 and has been validated against eight similar models and across nine culturally diverse nations. 32 It explains user behavior as being primarily a function of a person's intention to use and includes four predictive domains, described by Abbad (2007) as:

1. Performance expectancy (PE): “the degree to which an individual believes that using the system will help him or her to attain gains in job performance.” 31
2. Social influence (SI): “the degree to which an individual perceives that important others believe he or she should use the new system.” 31
3. Effort expectancy (EE): “the degree of ease associated with the use of the system.” 31
4. Facilitating conditions (FC): “the degree to which an individual believes that an organizational and technical infrastructure exists to support the use of the system.” 31

Gender, age, experience with similar technology, and voluntariness of use affect the impact of the predictive domains on BI. 31 For example, the effect of performance expectancy on behavior intention is modified by age and gender. 30

We sought to show that after a month of use, participants would report a high intention to use the application in the future and that the predictive UTAUT domains were positively associated with this intention. Based on instruments validated by Venkatesh et al., 30 33 a survey was developed to measure each of the UTAUT domains. 30 In total, 9 items were measured on a 5-point Likert scale (1 = strongly disagree, 5 = strongly agree). Items associated with particular UTAUT domains were averaged to give the score for that domain.

### Statistical Analysis

Statistical analysis was performed using STATA software, version 16.1. Continuous variables are shown as mean ± SD for normal distributions or median (interquartile range [IQR]) otherwise. We present categorical variables as *n* (%).

Baseline and 4-week average ACT scores were compared with the Wilcoxon signed-rank test. The change in the proportion of participants achieving good asthma control (ACT > 19) from baseline to week 4 was evaluated using McNemar's chi-square test. QoL scores at 2 and 4 weeks were compared using a paired *t* -test. The association between ACT score changes and BI (high/low and 1–5 scale) was analyzed using the Wilcoxon signed-rank test and Spearman correlation.

To assess determinants of strong intention to utilize ASTHMAXcelED, BI was categorized into agree/strongly agree versus neutral/disagree/strongly disagree. Spearman rank correlation assessed the link between performance expectancy, effort expectancy, social influence, and BI. Facilitating condition relates to actual use, not intended use, so it was excluded from our model. The association between BI with the other UTAUT domains, as well as other participant characteristics, was also measured using multivariable logistic regression. Age, sex, educational level, and UTAUT predictive variables were selected a priori for inclusion in this theory-informed model. Age and sex are included in the UTAUT framework, and education in years was selected as a proxy for experience in UTAUT. Interactions among independent variables were examined using product terms and stratified if clinically relevant with *p* < 0.25.

Missing ACT, QoL, and UTAUT responses were addressed using multiple imputation techniques. Continuous variables were imputed via predictive mean matching. Logistic regression was used to impute binary variables. The evaluation of imputations involved using distribution plots to compare complete, imputed, and observed data. Approximately 6% of the data was imputed. We also performed sensitivity analyses for the primary outcome and for the logistic regression model of BI using best- and worst-case assumptions.

## Results

### Characteristics of Study Participants

**Fig. 2:**
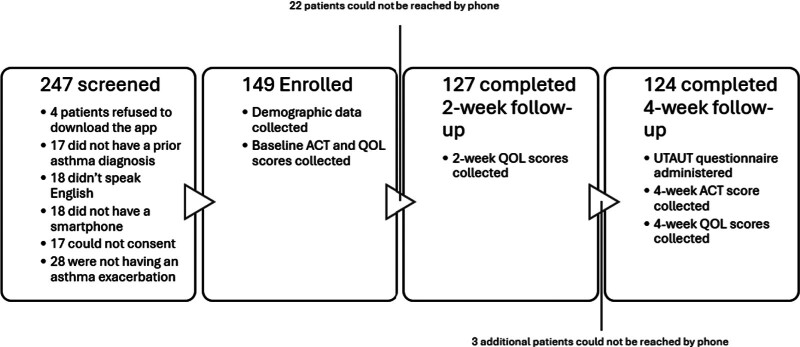
Patient recruitment approach flow chart. Created by Authors.

RAs screened 247 patients and enrolled 149 participants. A total of 127 participants completed 2-week follow-up; 124 completed 4-week follow-up ( Fig. 2 ). Of the patients not enrolled, 17 (17%) did not have a prior asthma diagnosis, 18 (18%) did not speak English, 18 (18%) did not have access to a smartphone, 17 (17%) were determined by the attending physician to be not medically/legally able to consent, and 28 (29%) were not diagnosed with asthma exacerbation. Our final analysis included the 124 subjects who completed the study. The median age of participants who completed the study was 37 years (IQR: 30, 48), with 77/124 (62%) being female, and 76/124 (61%) identifying as Hispanic. The median number of years of education was 12 (IQR: 4, 13) and the median number of ED visits and admissions for asthma in the past year was 1 (IQR: 0, 2) and 0 (IQR: 0, 0), respectively. A total of 58/124 (46%) patients were discharged on steroids. No significant differences existed between these participants and those lost to follow-up. A complete list of participant demographics and medical history can be found in Table 1 .

**Table 1:** Baseline characteristics of participants by initial level of asthma control

	ACT > 19 ( *n* = 34)	ACT ≤ 19 ( *n* = 90)	Lost to follow-up ( *n* = 25)
Age, y (median, IQR)	38 (31, 49)	36 (30, 47)	36 (29, 48)
Sex, *n* (%)			
Female	21 (62)	56 (62)	14 (56)
Male	13 (38)	34 (38)	11 (44)
Race, *n* (%)			
Non-Hispanic White	3 (9)	8 (9)	3 (12)
Non-Hispanic Black	12(35)	19 (21)	8 (32)
Hispanic	18 (53)	58 (64)	14 (56)
Asian/API	1 (3)	0 (0)	0
Other/decline	0 (0)	4(4)	0
Education, y (%)			
No primary education	0 (0)	2 (2)	0
Primary completed	1 (3)	2 (2)	1 (4)
Secondary completed	12 (35)	49 (54)	14 (56)
College completed	17 (50)	30 (33)	5 (20)
Graduate completed	4 (12)	7 (7)	4 (16)
ED visits, (median, IQR)	1 (0, 2)	1 (0, 2)	1 (0, 2)
Asthma admissions (median, IQR)	0 (0, 0)	0 (0, 0)	0 (0, 0)
Last visit with an asthma specialist, *n* (%)			
<1 mo ago	1 (3)	6 (7)	0
<6 mo ago	1 (3)	6 (7)	0
<1 y ago	3 (9)	7 (8)	1 (4)
>1 y ago	14 (41)	23 (26)	6 (24)
Never	15 (44)	48 (53)	18 (72)
Diabetes, *n* (%)	3 (9)	14 (16)	3 (12)
Hypertension, *n* (%)	6 (18)	14 (16)	3 (12)
Smoking status, *n* (%)			
Never smoked	24 (71)	47 (52)	13 (52)
Former smoker	4 (12)	15 (17)	5 (20)
Current smoker	6 (18)	23 (26)	7 (28)
Discharged with steroids, *n* (%)	10 (29)	48 (53)	11 (44)

Abbreviations: ACT, Asthma Control Test; API, Asian Pacific Islander; ED, emergency department; IQR, interquartile range.

### Asthma Control, Health Care Utilization, and Quality of Life Scores

The proportion of participants with good asthma control (ACT score > 19) significantly increased from 27% (34/124) to 54% (67/124), *p* < 0.01, 4 weeks after discharge ( Table 2 ). Participants with good asthma control did not have statistically different baseline characteristics than those with poor asthma control.

**Table 2:** Proportion of total participants with good asthma control

	Baseline, *n* (%)	Week 4, *n* (%)	*p*
ACT > 19	34 (27)	67 (54)	<0.01

Abbreviation: ACT, Asthma Control Test.

Median ACT score increased from 14 (IQR: 10, 20) to 20 (15, 24), *p* < 0.001 over 4 weeks. In the first 2 weeks after discharge, QoL scores for shortness of breath improved on average from 3.69 ± 2.03 to 4.82 ± 2.06, from 3.17 ± 2.13 to 4.45 ± 2.29 for dust in the environment, from 3.38 ± 2.05 to 4.95 ± 2.16 for wheeze, from 3.73 ± 2.31 to 4.64 ± 2.29 for weather or air pollution, and 3.95 ± 2.21 to 5.0 ± 2.21 for exercise tolerance. In weeks 2 to 4 after discharge, QoL scores for shortness of breath improved on average from 4.82 ± 2.06 to 5.15 ± 2.04, from 4.45 ± 2.29 to 4.87 ± 2.21 for dust in the environment, from 4.95 ± 2.16 to 5.26 ± 2.11 for wheeze, from 4.64 ± 2.29 to 5.06 ± 2.24 for weather or air pollution, and 5.0 ± 2.21 to 5.33 ± 2.05 for exercise tolerance ( Table 3 ).

**Table 3:** Asthma Control Test and quality of life scores 4 weeks after discharge

	ACT	Shortness of breath	Bothered by dust in the environment	Experience wheeze	Bothered by weather or air pollution	Limited by activity
Baseline	14 (10–20)	3.69 ± 2.03	3.17 ± 2.13	3.38 ± 2.05	3.73 ± 2.31	3.95 ± 2.21
2-wk follow-up	–	4.82 ± 2.06 a	4.45 ± 2.29 a	4.95 ± 2.16 a	4.64 ± 2.29 a	5.0 ± 2.21 a
4-wk follow-up	20 (15, 24) b	5.15 ± 2.04 c	4.87 ± 2.21 c	5.26 ± 2.11	5.06 ± 2.24	5.33 ± 2.05

Abbreviation: ACT, Asthma Control Test.

a Difference between baseline and week 2 significant at *p* < 0.05 level.

b Difference between baseline and week 4 significant at *p* < 0.05 level.

c Difference between week 2 and week 4 significant at *p* < 0.05 level.

After 4 weeks, 9/124 (7%) patients reported having returned to any ED for asthma during the study period. No patients reported being hospitalized for asthma ( Table 4 ). There were no significant differences in baseline characteristics between the two groups.

**Table 4:** Frequency of emergency department revisit/hospitalization within 4 weeks of discharge

ED revisit for asthma within 4 wk, *n* (%)	Hospitalization for asthma within 4 weeks, *n* (%)
9 (7)	0 (0)

Abbreviation: ED, emergency department.

### User Acceptance and Intention to Use

A total of 48/122 patients (39%) gave ASTHMAXcelED positive BI to use scores, whereas 6/122 (5%) gave the app negative scores. Two patients stated they never used the app at all, and 68/122 had a neutral response. Univariate analysis revealed that performance expectancy, effort expectancy, and social influence were positively associated with BI ( Table 5 ). After adjusting for age, sex, and level of education, performance expectancy and effort expectancy remained positively associated with BI ( *p* < 0.02). None of the effect modifiers in the UTAUT model (age, education) had significant interactions with the main predictive variables of UTAUT. The multivariable logistic regression model achieved a McFadden's pseudo-R ^2^ of 0.72 and c-statistic of 0.98 ( Table 6 ).

**Table 5:** Univariate relationships of predictive Unified Theory of Acceptance and Use of Technology domains with behavioral intention

	Behavioral intention to use score ( *n* = 123) a					
Domains (median, IQR)	1 ( *n* = 5)	2 ( *n* = 1)	3 ( *n* = 68)	4 ( *n* = 24)	5 ( *n* = 24)	rho
Performance expectancy (PE) a	2 (1, 2)	3	3 (3, 3)	4 (4,4)	4.3 (4, 5)	0.80 ( *p* < 0.001)
Effort expectancy (EE) a	3.5 (3.0, 4.0	3.5	3 (3, 3)	4 (4, 4.3)	5 (4.8, 5)	0.72 ( *p* < 0.001)
Social influence (SI) a	1 (1, 3)	3	3 (3, 3)	4 (4, 4)	4 (3, 4.5)	0.69 ( *p* < 0.001)

Abbreviation: IQR, interquartile range.

a Missing responses due to patients stating they did not use the app).

**Table 6:** Multivariable regression model of predictors of behavioral intention a , b

	OR	β	95% CI	*p*
Performance expectancy	6.85	1.92	0.31–3.54	0.02
Effort expectancy	11.22	2.42	0.92–3.91	0.002
Social influences	10.05	2.31	0.67–3.95	0.006
Age (y)	10.3	0.03	−0.03 to 0.10	0.32
Educational level (y) a	0.92	1.32	−0.26 to 0.08	0.32
Sex	3.74	−0.09	−3.62	0.15

Abbreviations: CI, confidence interval; OR, odds ratio.

a Pseudo-R ^2^ = 0.72, C-statistic 0.98.

b Missing value computed with multiple imputation.

### The Association between Asthma Control and Intention to Use ASTHMAXcelED

The average change in ACT score was higher among users with a higher BI score. Participants who agreed/strongly agreed that they would use ASTHMAXcelED in the next 6 months (BI 4 and 5) had an average ACT score improvement greater than 3 (4.10 ± 7.49), whereas patients who disagreed/strongly disagreed (BI 1 and 2) had an average change in ACT score less than 3 (2.5 ± 7.9). Participants with a neutral response had an average change in ACT score of 3.78 ± 6.39. This relationship was not statistically significant The relationship between BI and change in ACT score over the study period is summarized in Table 7 .

**Table 7:** Association between Asthma Control Test (ACT) and Behavioral Intent

	Behavioral Intent score ( *n* = 122)		
	Negative	Neutral	Positive
ACT change	2.5 ± 7.9	3.78 ± 6.39	4.10 ± 7.49

## Discussion

Our study demonstrated that in the four weeks after introduction to the app, participants had sustained improvement in asthma symptom control and asthma-related QoL. The median ACT score across the 4 weeks increased by 6.0 points, thereby surpassing the ACT's threshold for clinical significance (change in ACT score of at least 3). All QoL scores improved from the baseline visit to the first follow-up at week 2, and continued to improve from week 2 to week 4 across all domains. These findings are promising as they suggest that ASTHMAXcelED could potentially deliver sustained improvements in asthma control and QoL. The improvements in ACT and QoL score reported in this study are larger than seen in prior versions of ASTHMAXcel. 34 Our cohort likely had more severe asthma that led to their presentation to the ED and explains the larger improvement in asthma control and QoL. A Cochrane review of smartphone and tablet asthma self-management apps reported that few preexisting mobile applications showed improvement in asthma symptom control, and that the effect of apps on asthma related QoL was inconsistent. 35

Building on the previous success of ASTHMAXcel in the clinic setting, our ED-based implementation shows that our approach can improve patient outcomes across different outpatient populations. One prospective cohort study of ED patients who received a different smartphone application achieved an improvement in ACT score of 4.5 in the postdischarge period. 36 Participants in our study had an ACT improvement of 6.0; while further research is needed, these results are promising. Additionally, the rate of steroid prescription upon discharge in this cohort was only 46%; due to the short-term impact of steroids primarily in the immediate postdischarge period, the impact of ASTHMAXcelED as an intervention is less likely to be confounded by concurrent medication use. No patients in this study were started on inhaled corticosteroids, which is in line with previous studies showing that patients discharged from the ED are rarely started on controller medications. 37 Additionally, the duration of action of prednisone is 12 to 36 hours and is therefore unlikely to produce continued improvements in asthma-related outcomes in the 2- to 4-week window after discharge. 38 The sustained improvement beyond the immediate postdischarge period, particularly in QoL scores between weeks 2 and 4 supports the conclusion that ASTHMAXcelED promoted sustained improvement in asthma-related patient-reported outcomes.

The ED revisit rate in this study was lower than expected, particularly when taking into account the demographics of our study population. ED revisits for asthma vary by race; one multicenter 2017 study noted that, in the absence of specific interventions to address asthma, Black patients had an ED revisit rate of 16.4%, and Hispanic patients had a revisit rate of 14.6%, compared with 12.5% among Whites and 5.1% among Asians. 39 Our cohort was 87% Hispanic or Black, and achieved a revisit rate of 7%. The ED revisit rate achieved by our cohort was also slightly lower than has been reported in similar populations, including a New York City public hospital survey that reported an ED revisit rate of 9.3%. 40 Another randomized study of a different application showed no improvement in ED revisits and hospitalization for asthma after app use. 41 ASTHMAXcelED and its tailored content may have contributed to this comparatively lower revisit rate.

What differentiates ASTHMAXcelED from other mobile applications is that its guideline-based educational content was up to date, was associated with improvement in patient-centered clinical outcomes, was a user-centered design intended for ED patient use, and was received positively by users. In Tinschert's review of 38 apps related to asthma self-management, none had been studied to determine their effect on clinical outcomes. 22 Additionally, many applications geared toward asthma patients are either not studied for user acceptance or have poor user feedback. 22 23 Very few study participants gave ASTHMAXcelED PE, EE, and BI scores less than 3, suggesting the application is an easily adopted and well-received form of self-directed patient education. While vastly more users gave ASTHMAXcelED, a positive intention to use score rather than a negative score, most users gave it a neutral score. User engagement is a challenge for almost all health apps 42 and a future study should explore strategies to improve user engagement in this population to improve its responsiveness to user needs and preferences. Patients with greater ACT score changes reported a stronger desire to continue to use the app, indicating that patients themselves were able to perceive the improved asthma symptom control that resulted from app usage. Their receptiveness to the app demonstrates the ease of use and ease of integration into their daily routine, as they likely deemed this app a helpful addition to their asthma regimen. These scores suggest that ASTHMAXcelED's user-centered design allowed it to meet the needs of the ED asthma population. While the differences were not statistically significant, the study was not powered to detect differences in this outcome, and the trend observed warrants further study.

This study had several limitations. As a nonrandomized, unblinded prospective cohort study, clinical improvement cannot be definitively attributed to the effect of ASTHMAXcelED. The study was performed in a single center whose demographics differ from the United States and only enrolled English-speaking patients. Care should be taken when generalizing these results to other populations. Nevertheless, the recruitment of Black and Hispanic patients is important given their historic underrepresentation in mHealth-related studies. Attrition from the study may introduce bias; however the lack of baseline differences is reassuring. Additionally, while interactions among independent variables were examined using product terms and stratified if clinically relevant with *p* < 0.25, future work should ensure that a theory-driven approach is taken to examine interactions to avoid inflation of type 1 error rates.

## Conclusion

The ASTHMAXcelED application was intentionally designed to be a self-directed form of asthma education that could assist patients in asthma self-management upon discharge from the ED. Users experienced sustained improvement in asthma control and QoL during the study period. Next steps include testing additional personalized features such as patient-reported outcomes, medical record integration, and non-English languages, as well as a randomized trial comparing the addition of an iteratively improved ASTHMAXcelED application to standard discharge protocols.

## Notes

Clinical Relevance Statement Use of ASTHMAXcelED was associated with a more than doubling of the proportion of adults achieving sustained asthma control improvements in the 4 weeks after discharge from the ED for an asthma exacerbation, with few return visits and hospitalizations. Combined with the overall high user acceptance scores, these findings suggest that integrating a user-centered mHealth tool into routine ED workflows can support evidence based self-management education past the bedside, which is particularly relevant for socioeconomically disadvantaged populations. It represents a scalable efficient follow-up support solution that improves patient outcomes without adding to staff burden.

## Notes

Multiple-Choice Questions

1. Which component of the ASTHMAXcelED app was designed to reinforce medication adherence and trigger avoidance?
  - Nine guideline bullet notes embedded in the app.
  - Personalized push notifications timed to patient medication doses.
  - Biweekly push notifications with best-practice tips.
  - Chatbot functionality to respond to individual user questions.
  **Correct Answer** : The correct answer is option c. ASTHMAXcelED sends concise, one-sentence push notifications biweekly. These messages included environmental trigger avoidance and medication adherence reminders, which were derived from prior versions of ASTHMAXcel to maximize engagement without creating notification fatigue.
2. The study's inclusion of which participant demographic strengthens its clinical relevance for underserved populations?
  - Predominantly non-Hispanic White adults.
  - Predominantly rural older adults.
  - Predominantly Hispanic and Black urban adults.
  - Predominantly female patients.
  **Correct Answer** : The correct answer is option c. Eighty-seven percent of the cohort identified as Hispanic or Black. They represent populations that are historically underrepresented in clinical trials generally and mHealth specifically while simultaneously having a disproportionately high asthma burden.
3. According to the authors, what key step is required prior to widescale rollout of ASTHMAXcelED?
  - Eliminating push notifications to avoid alert fatigue.
  - Conducting a randomized trial after adding personalization and non-English content.
  - Replacing all standard discharge instructions with the app in the EMR.
  - Adding more detailed educational content.
  **Correct Answer** : The correct answer is option b. The authors call for testing additional personalized features, EMR integration, and multilingual modules in a randomized trial comparing an adapted ASTHMAXcel app against usual discharge protocols. This will definitively establish effectiveness and generalizability, prior to full implementation.
